# Identification of regulatory structure and kinetic parameters of biochemical networks via mixed-integer dynamic optimization

**DOI:** 10.1186/1752-0509-7-113

**Published:** 2013-10-31

**Authors:** Gonzalo Guillén-Gosálbez, Antoni Miró, Rui Alves, Albert Sorribas, Laureano Jiménez

**Affiliations:** 1 Departament d’Enginyeria Química, Universitat Rovira i Virgili, Av.Països Catalans 26, 43007 Tarragona, Spain; 2 Departament de Ciències Mèdiques Bàsiques, Institut de Recerca Biomèdica de Lleida (IRBLLEIDA), Universitat de Lleida, Avinguda Alcalde Rovira Roure 80, 25198 Lleida, Spain

**Keywords:** Parameter estimation, Structure identification, Akaike criterion, Orthogonal collocation, Dynamic optimization, Biochemical networks

## Abstract

**Background:**

Recovering the network topology and associated kinetic parameter values from time-series data are central topics in systems biology. Nevertheless, methods that simultaneously do both are few and lack generality.

**Results:**

Here, we present a rigorous approach for simultaneously estimating the parameters and regulatory topology of biochemical networks from time-series data. The parameter estimation task is formulated as a mixed-integer dynamic optimization problem with: (i) binary variables, used to model the existence of regulatory interactions and kinetic effects of metabolites in the network processes; and (ii) continuous variables, denoting metabolites concentrations and kinetic parameters values. The approach simultaneously optimizes the Akaike criterion, which captures the trade-off between complexity (measured by the number of parameters), and accuracy of the fitting. This simultaneous optimization mitigates a possible overfitting that could result from addition of spurious regulatory interactions.

**Conclusion:**

The capabilities of our approach were tested in one benchmark problem. Our algorithm is able to identify a set of plausible network topologies with their associated parameters.

## Background

Mathematical models of biochemical systems are becoming essential in systems biology to complement and extract information from time series. This information can be of two types. On the one hand, if the structure of the molecular circuit that executes the process of interest is known, models can be used to infer the numerical parameters that govern the dynamics of the system [1-4]. On the other, models can be used to infer the structure of the system from time series data (see for example [5-7]).

In either case, to obtain a useful model, we face different challenges: (i) defining the system’s mass flow structure (*stoichiometry*), (ii), deciding the appropriate mathematical representation (*kinetics*), (iii) estimating the parameters that make the model response consistent with experimental data (*parameter estimation*), and (iv) inferring the system’s regulatory structure. In addition, once the model is well defined, it should be able to predict systemic responses under yet untested experimental conditions (*model validation*).

The four challenges described in the previous paragraph are often addressed in independent steps. Current solutions to the first challenge are generally based on compiling information about the system and using that information to create the stoichiometric matrix for the system one wants to analyze (see for instance [8]). To solve the second challenge we need to define kinetic functions that describe the dynamic behavior of the dependent variables of the system. If the kinetic functions are unknown, approximate formalisms that have a solid theoretical support can be used to describe the dynamic behavior of the system within a given accuracy [9,10]. The third challenge is typically formulated as an optimization problem that minimizes the sum of squared residuals between the measured and simulated data (see a review of methods in [1]). The type of optimization problem being faced and the technical challenges to be solved depends upon the biological model of choice, upon the experimental data available, and upon the specific mathematical formalism used [11,12]. In many practical applications, the target biological system is described through nonlinear ordinary differential equations (ODEs). Hence, the parameter estimation task gives rise to dynamic optimization problems that are hard to solve. The fourth challenge could in principle be addressed in the same way as the first. However, despite the enormous amount of biological information available in public databases, regulatory signals are, in general, poorly understood and hardly ever properly characterized *in vivo*. Regulatory signals appear in a model as parameters accounting for the influence that metabolites others than the substrates of a reaction have on its velocity. Hence, parameter fitting can also be used to address the fourth challenge. However, the overwhelming majority of parameter estimation methods assumes a given structure and considers a fix regulatory scheme (see a review in [1]). This simplification is motivated by the difficulty in identifying regulatory effects, a task for which a myriad of alternative kinetic models must be explored [7,13-15].

Traditional methods for the selection of biological systems have mostly applied regression or chi-squared-based criteria (rather than information-theoretic fit criteria) [16]. However, information-theoretic criteria such as the Akaike’s Information Criterion (AIC) [17] or the Bayesian Information Criterion (BIC) [18], are now perceived as important measures to assess quality of fit. AIC is often preferred over BIC becaue it has a more immediate connection to the theory of information [19]. AIC captures the trade-off between the complexity (measured by the number of parameters), and accuracy of the fitting. Smaller AIC values imply a better approximation to the model sought.

In this work we propose a strategy to simultaneously address the four challenges described above that relies on the use of mixed-integer dynamic optimization (MIDO) methods. Our approach adopts a structured mathematical framework to represent the kinetics of the processes that is flexible enough to reproduce a set of plausible network topologies (by implementing slight modifications on a basic model formulation). The power-law [20] and the saturable and cooperative formalisms are examples of such general kinetic representations [9]. Based on this type of general kinetic modeling framework, we develop our systematic parameter estimation method that provides as output a set of potential reaction and regulatory topologies for the target network along with the associated model parameters. We illustrate the capabilities of our approach using the GMA kinetic representation, a canonical model structure that uses the power-law kinetic formalism [21,22].

## Results and discussion

**Figure 1:**
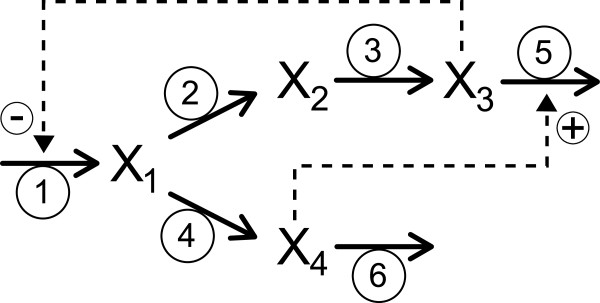
**Reference system taken from Voit and Almeida **[23]**(default parameters are shown in Table **1**).**

As a proof-of-concept, we have tested the capabilities of our approach through its application to a case study taken from Voit and Almeida [23]. The system considered is a four-constituent pathway branched with six velocities and two regulatory signals. *X*_*1*_ is generated from *X*_*0*_, and its production is inhibited by *X*_*3*_ which is produced from *X*_*1*_ via intermediate *X*_*2*_. *X*_*1*_ yields also *X*_*4*_, which promotes the degradation of *X*_*3*_ (see Figure 1).

### Parameter estimation when the regulatory structure is known

We shall first show that the proposed method is capable of appropriately identifying the model parameters using dynamic data when the regulatory structure is known. This is the classical parameter estimation problem that is solved in many applications. To this end, we first produce dynamic data without error from the reference system using a specific set of parameter values. Then, this *in silico* data is labeled as experimental and we use the proposed method to estimate the model parameters. We define a dynamic optimization model that contains a set of dynamic differential equations describing the system’s kinetics. This dynamic model is reformulated into a nonlinear program (NLP) using orthogonal collocation on finite elements. This NLP does not contain binary variables because we assume that the regulatory signals are known. The aforementioned NLP was implemented in GAMS 23.7.3 and calculated with CONOPT 3.15A on a PC/AMD Athlon at 2.99 Ghz using a single core. The NLP features 302 variables and 285 constraints, and was solved in 2.3 CPU seconds. As expected, we obtain estimated parameters values that are very close to the original ones (see Table 1), and a least square error of 1.45 × 10^-6^.

**Table 1:** Original and predicted parameters values

**Parameter**	**Original parameters**	**Proposed algorithm**
*f*_13_	-0.8	-0.7999
*f*_21_	0.5	0.4996
*f*_32_	0.75	0.7494
*f*_41_	0.5	0.5006
*f*_53_	0.5	0.4996
*f*_54_	0.2	0.1996
*f*_64_	0.8	0.8010
γ_1_	12	12.000
γ_2_	8	8.0031
γ_3_	3	3.0034
γ_4_	2	1.9965
γ_5_	5	5.0014
γ_6_	6	5.9967

Data is error free (one experiment with only one observation by time point).

**Figure 2:**
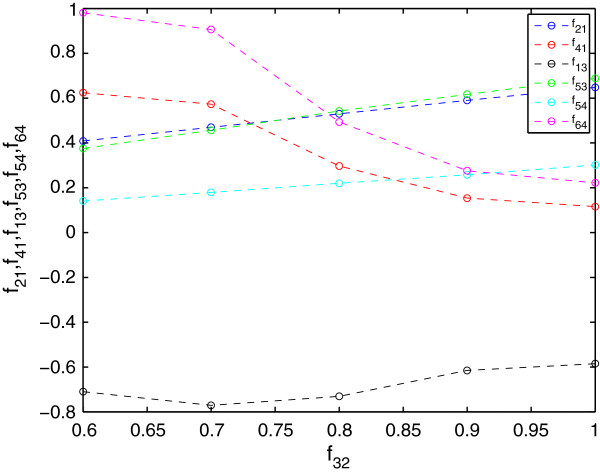
**Values of the fitted parameters for different values of f32.** Each point was generated by fixing f32 and solving the NLP free of error.

Non-linear kinetic models, like the GMA representation, have a certain degree of plasticity that allows different parameter sets to fit the same data. Clear parameter trends are obtained by fixing a given parameter and fitting the remaining ones. As an example, Figure 2 shows the results of fixing *f*_*32*_ at different values and fitting the other parameters. All the points in the figure lead to residuals below 5.88 × 10^-4^, indicating that it is possible to obtain good fits with different parameter sets. Similar patterns are obtained if we choose to fix any other parameter of the set.

As observed, the model is rather flexible, as there are many combinations of parameters values leading to very low residuals and essentially the same fit to the data. In practical terms, this means that given an experiment and an estimation procedure, we could obtain different parameter sets that closely reproduce the experimental measurements, but that differ from the actual values with which the dynamic profile was generated *in silico*. Thus, estimated parameter values don’t help comparing the obtained fit with the reference model. In practice, the residual error and the resulting time profiles should be used to assess the fit.

**Figure 3:**
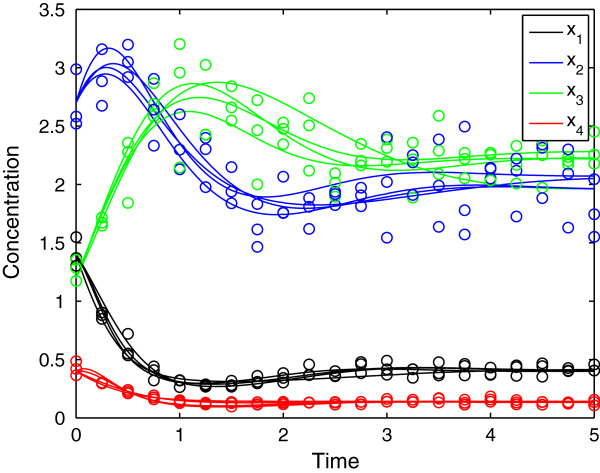
Adjusted profiles for four different noisy data sets (i.e. one experimental condition and four replications) with a standard deviation of 10%.

We will now consider the effect of noisy data on fitting the model, as such noise plays a key role in evaluating any proposed method for identifying the regulatory structure of a network. To explore the influence of random experimental uncertainty, we generated 100 dynamic profiles from the reference model by introducing statistical noise. For this, we applied Monte Carlo sampling assuming that every data point follows a normal distribution with standard deviation values of 0.5, 1, 5 and 10% of the actual nominal value. For comparison purposes, we use the same perturbation experiment as in the previous example. Table 2 shows the parameter values and the associated residuals obtained for four of the samples generated, while Figure 3 depicts the profiles associated with a standard deviation of 10%. We can appreciate that despite the different parameter values, the various fitted models lead to similar residuals. Note that although the regulatory structure is fixed, we obtain parameter values representing either positive or negative regulatory effects (*f*_*54*_) of *X*_*4*_ on *v*_*5*_. This is a consequence of the “experimental error” introduced in the noisy data. That error may force the estimation procedure to an optimum involving a set of parameter values that may be different from the set that generates the noiseless data. In addition, as seen above, different parameters sets can be used to produce similar time courses. This means that there are coupled parameters in the system, which may also contribute for the estimation of regulatory interactions with reversed signals.

**Table 2:** Parameters values with noisy data (one experiment)

		**10%**		
	**Profile 1**	**Profile 2**	**Profile 3**	**Profile 4**
*f*_13_	-0.14	-0.27	-0.84	-0.79
*f*_21_	0.26	0.47	0.4	0.29
*f*_32_	0.44	1	0.64	0.41
*f*_41_	0.04	0	0.9	1
*f*_53_	0	0.26	0.42	0.12
*f*_54_	-0.06	0.04	0.1	-0.12
*f*_64_	0.13	0.07	1	1
Residual	1.88	1.67	1.68	2.29
		**5%**		
	**Profile 5**	**Profile 6**	**Profile 7**	**Profile 8**
*f*_13_	-0.282	-0.532	-0.631	-0.893
*f*_21_	0.56	0.618	0.306	0.6
*f*_32_	1	1	0.436	1
*f*_41_	0	0.092	0.761	0.742
*f*_53_	0.368	0.639	0.273	0.298
*f*_54_	0.127	0.244	0.021	0.279
*f*_64_	0.064	0.158	1	1
Residual	0.4128	0.4203	0.5706	0.4482
		**1%**		
	**Profile 9**	**Profile 10**	**Profile 11**	**Profile 12**
*f*_13_	-0.881	-0.427	-0.859	-0.71
*f*_21_	0.571	0.523	0.5	0.414
*f*_32_	0.885	0.809	0.758	0.608
*f*_41_	0.587	0.078	0.661	0.656
*f*_53_	0.479	0.467	0.507	0.402
*f*_54_	0.2	0.176	0.197	0.136
*f*_64_	1	0.162	1	1
Residual	0.0207	0.0163	0.0167	0.0227
		**0.5%**		
	**Profile 13**	**Profile 14**	**Profile 15**	**Profile 16**
*f*_13_	-0.845	-0.744	-0.843	-0.765
*f*_21_	0.535	0.472	0.496	0.453
*f*_32_	0.816	0.714	0.749	0.673
*f*_41_	0.556	0.492	0.647	0.643
*f*_53_	0.492	0.439	0.497	0.443
*f*_54_	0.201	0.167	0.196	0.164
*f*_64_	0.916	0.816	1	1
Residual	0.0052	0.0041	0.0042	0.0057

We solved a total of 100 problems, each corresponding to a different replication, generated randomly see Additional file 1: Table S1). The table shows the 16 cases for which the residual error is low.

In general, even in simple cases as the one considered here, it will be difficult to obtain a consistent estimation from a single time-series. Identifying the parameter set that is more likely to be the correct one requires simultaneous fitting to additional time-series, resulting from more than one set of experiments. By doing so, we will constraint further the admissible parameter sets (see [24]). In Table 3, we show the results of fitting three different experiments with experimental error. Each experiment corresponds to an alternative perturbation on the initial concentration of metabolite *X*_*3*_ (0.2, 1.2, and 2.2). These perturbations force the system to move across different dynamic regimes, producing additional information that helps in the identification of appropriate parameter values. As observed, the estimated parameters are more consistent over the various experiments. They are also closer to the actual parameter set selected for generating the data. Note, however, that it is still possible to find solutions involving alternative regulatory topologies with good fit to data (*f*_54_ acting as an inhibitor in Profile 2).

**Table 3:** Parameter values obtained from simulated noisy data (with noisy data (three experiments))

	**Profile 1**	**Profile 2**	**Profile 3**	**Profile 4**
*f*_13_	-0.67	-0.64	-0.62	-0.92
*f*_21_	0.33	0.9	0.49	0.69
*f*_32_	0.42	1	0.73	1
*f*_41_	0.64	0	0.38	0.26
*f*_53_	0.49	0.66	0.3	0.4
*f*_54_	0.05	-0.95	0.22	0.34
*f*_64_	1	1	0.53	0.58
Residual	6.96	7.10	5.39	4.89

We solved a total of 100 problems, generated randomly. See Additional file 1: Table S2.

### Identifying the regulatory structure

#### Performance using error free data

After testing the capabilities of the method when the structure is known, we studied its ability to identify the regulatory topology of the model. To this end, we explore the performance of the method using one experiment with low experimental error (i.e., assuming that the data follow normal distributions with a standard deviation of 0.5%). Larger errors result in a wider set of alternative structures and for simplicity’s sake we shall not discuss them here.

In order to simplify the search, we fix a maximum of two metabolites (the substrate of the reaction, which is given by the stoichiometric information, and one possible additional modifier, which is not *a priori* characterized) as potential variables affecting each velocity.

We note that it is typical to have some *a priori* knowledge about the biological system one is interested in. The complexity of the regulatory interactions in the identification problem is reduced if such knowledge can be used to constrain further both, the number of potential regulatory signals in the model and their signs (positive, negative). In such cases, we can introduce specific constraints for the relevant parameters to be fitted. For example, in our case kinetic-order corresponding to the substrates of a reaction must be positive.

The MINLP model that simultaneously fits the parameters and infers probable regulatory interactions was implemented in GAMS 23.7.3 and solved with the solver SBB in the same computer as before. The model has 72 binary variables, 391 continuous variables and 414 equations. The solution time was in the order of few minutes for each simulation.

**Figure 4:**
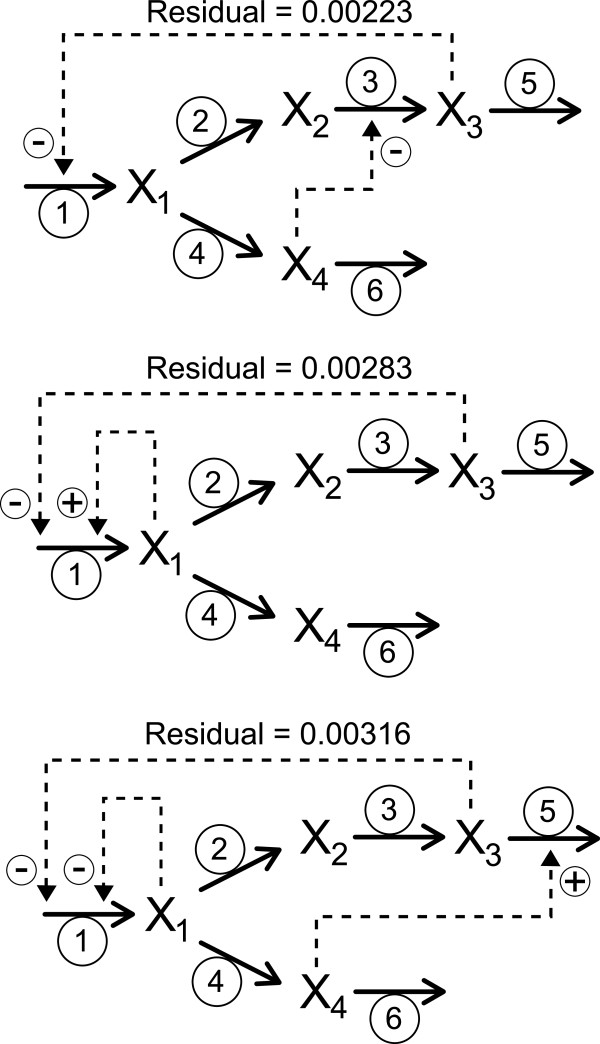
**The proposed method identifies different regulatory topologies that essentially produce the same output.** We show here the associated profiles corresponding to three regulatory structures with lowest residual values obtained by analyzing data from a single experiment with one replicate (see parameters values and residuals in Additional file 1: Table S3.

**Figure 5:**
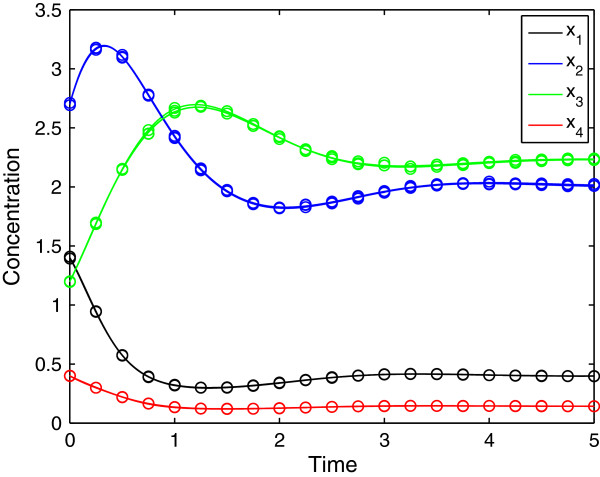
**Dynamic responses corresponding to the three different topologies of Figure **4**.** Parameter values are indicated on Additional file 1: Table S3.

Our algorithm identifies a set of compatible systems, since the model has enough flexibility to play with the regulatory structure as well as the kinetic parameters when minimizing the residuals. The method identifies topologies that are quite close and that show very small residuals, but it is unable to uniquely identify the original topology (see Additional file 1: Table S3 for a list of topologies generated and their associated kinetic parameters and residuals). As an example, in Figure 4, we compare three completely different regulatory structures that produce almost indistinguishable results and similar fitting to the actual dynamics, leading to residual values of 0.00223, 0.00283 and 0.00316 (Figure 5).

As before, one strategy for increasing the possibility of correctly identifying the “true” regulatory structure is to use additional time-series data of the same system under different sets of initial conditions. To this end, we changed the initial concentrations of *X*_*3*_ (0.2, 1.2, and 2.2). The MINLP model was again implemented in GAMS and solved with SBB in the same computer. In this case, the MINLP features 72 binary variables, 967 continuous variables and 980 equations. The solution time was in the order of few minutes for each simulation.

**Figure 6:**
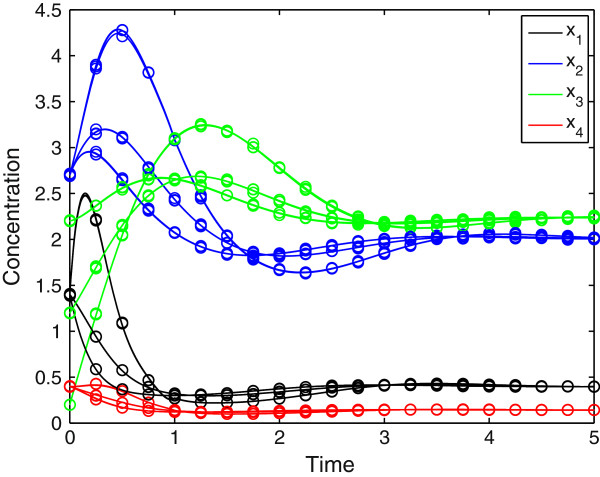
**The Profiles generated from three different topologies and three experiments with one replication each.** The experiments are generated from the base case by applying different perturbations in the initial concentration of *X*_*3*_. Details on the topology and associated parameters are provided on Additional file 1: Table S4.

In Figure 6 we show the dynamic profiles associated with three different topologies identified by the MINLP. A complete list of network topologies and associated kinetic parameters and residuals is provided as (Additional file 1: Table S4). With three time series, the method identifies not only the actual topology, but also several structures that contain the original one (i.e., topologies that account for all the actual regulatory effects plus other signals that were not present originally). Again, we obtained slightly different parameter sets in each case, since the model flexibility is rather large.

### Additional remarks

The use of MIDO techniques combined with orthogonal collocation allows posing the parameter estimation task as an algebraic optimization problem that can be efficiently solved using standard MINLP algorithms. Orthogonal collocation shows some appealing properties (see [25]), but has the drawback of increasing the model size because it adds auxiliary variables and equations that increase the problem complexity. Our MIDO approach, however, can be solved by any MIDO algorithm, and it is not restricted to the use of orthogonal collocation and MINLP reformulations.

A key point in our method is the selection of an appropriate starting point to initialize the MINLP algorithm. Standard MINLP algorithms typically solve an initial NLP where the binary variables are relaxed. If this NLP does not converge, the entire algorithm might fail. An initialization strategy that works well in practice is to integrate first the original kinetic model for some parameter values, and then use the dynamic profiles generated *in silico* to provide a starting point for the NLP solver. Another method consists of solving an auxiliary model where we relax some constraints through the addition of slack variables, and then minimize the summation of the slacks in order to obtain an initial feasible point. With this relaxed model, we can identify a feasible (but not necessarily optimal) solution for the initial NLP.

Even if the MINLP model converges, there is still the issue of getting trapped in local optima during the search. To avoid this, we can run the optimization algorithm from different starting points generated randomly. This strategy does not guarantee convergence to the global optimum, but tends to produce high quality solutions in short CPU times. In contrast, deterministic global optimization methods provide a rigorous interval within which the optimum should fall, but tend to lead to large CPU times (see [26,27]).

In our case, we initialize the NLPs by solving a set of relaxed problems from different starting points and then pass these results to the first NLP solver. This approach provides feasible points from which the model converges to solutions with low residuals.

In general, due to the nonconvex nature of the reformulated MINLP, the nonlinear branch and bound implemented in SBB outperforms the outer-approximation used by DICOPT. This is because the supporting hyperplanes defined in the master MILP solved by DICOPT may chop-off feasible solutions due to the noconvex nature of some nonlinear inequalities.

We note that nonlinear models are hard to handle, and even more so when they contain binary variables. Standard NLP solvers can solve problems containing up to hundreds of thousands of variables and constraints. On the other hand, the computational burden of MIDO (and MINLP) models is rather sensitive to the number of binary variables. For the type of problems we are dealing with, it is difficult to provide a bound on the number of binaries above which the algorithm might fail. In practice, however, we found that this approach efficiently for less than one hundred binaries (around 30 parameters).

From a practical viewpoint, we face the challenging problem of discriminating between compatible regulatory structures for a given data set. On a worst case scenario, our method provides a ranked set of alternative regulatory topologies that can be tested and validated experimentally. If appropriate additional time-series data are available, the set of admissible solutions for testing can be further constrained and reduced. Our method finds a set of alternatives that are consistent with the dynamic data available and that can be further refined using additional information and expert knowledge on the system. (i.e., complementary biological information). For instance, kinetic-orders that correspond to substrates of a reaction may be safely restricted to be positive. Similarly, if we are fairly sure that a given metabolite does not participate in a reaction, its kinetic-order should be fixed to zero.

Our method can also be used to explore hypotheses about the regulatory structure of a system. For instance, we can force some parameters to take negative values, thereby representing inhibition effects, and then perform the optimization so as to determine if the fitting is good enough. Furthermore, we can follow the same procedure in order to identify regulatory effects that are consistent with this hypothesis.

In addition, we note that our approach can be easily adapted in order to work with other model selection criteria besides AIC. We remark, however, that the assessment of different selection criteria would deserve a comprehensive study that is beyond the scope of this work.

The simple examples presented in this paper show that estimating parameters in dynamic kinetic models is far from being an easy job and that models based on the power-law formalism facilitate the estimation task. Although this formalism is suitable for a wide variety of problems, one may argue that it may present some limitations. As an alternative, we can use extensions of this framework such as the Saturable and Cooperative formalism [9], which takes into account saturation effects. In both cases, a key point is the possibility of using a canonical mathematical formalism that facilitates the automatic search of alternative regulatory patterns. The method described here would be applicable to such models via recasting of the Saturating and Cooperative formalism into a power law [28].

## Conclusions

In this work we have proposed a rigorous approach based on mathematical programming for the simultaneous identification of the regulatory signals and estimation of the kinetic parameters of models of biochemical networks. Our approach is based on the use of mixed-integer dynamic optimization (MIDO) models that minimize the Akaike criterion, and that can be solved by standard optimization algorithms. Particularly, we solve this MIDO by reformulating it as a mixed-integer nonlinear program (MINLP) using orthogonal collocation on finite elements, which makes it possible to apply standard MINLP solution algorithms in an iterative fashion in order to identify a set of plausible network topologies and associated kinetic parameters.

It is noteworthy that the difficult task of parameter estimation in nonlinear models becomes really complicated as the size of the models increases. Therefore, such estimation typically requires customized solution procedures. One key point is to use the appropriate initial conditions to ensure convergence of the calculations.

The proposed method can contribute to fill the lack of information on the regulatory signals that are in play in a given metabolic scenario. Although we cannot deal with genome-wide models, we have shown that dynamic profiles can be processed to provide clear hypothesis on the underlying regulatory structure. This is an important step towards completing essential information on different metabolic processes that are poorly understood.

## Methods

The problem we address here is to infer the regulatory structure of a metabolic system, given a known structure for the reaction network (stoichiometry) and experimental time series for the dynamic behavior of that system. To address this question, and to explore the practical problems associated, we consider the following general representation of a biochemical network:

(1) $${\dot{X}}_{i}=\sum_{r=1}^{p}\mu_{i,r}\upsilon_{r\;}i=1,\ldots ,n$$

where *X*_*i*_ denotes the concentration of metabolite *i*, *μ*_*i,r*_ is the stoichiometric coefficient of metabolite *i* in process *r*, which indicates the number of molecules of type *i* produced or destroyed by process *r*, and *v*_*r*_ is the rate function of this process. In general, *v*_*r*_ is represented as:

(2) $$\upsilon_{r}\left(X_{1},\ldots ,\;X_{n+m},\;\theta \right)$$

There are two critical issues in defining this model. One is the selection of an appropriate mathematical representation for *v*_*r*_, which may be a function of an arbitrary number of variables (substrates, products, and modifiers). In most cases the mechanism for each process are unknown and choosing a specific mechanistic rate law, such as a Michaelis-Menten rate law, becomes an act of faith. The other issue is the problem of identifying the regulatory structure of the system.

The most straightforward and theoretically well supported solution to both issues is the use of an approximate formalism based on a standard mathematical representation [10]. By adopting such a kinetic representation, identifying the regulatory structure of the system becomes synonymous to determining the set of values *θ* for the model parameters that better fit the available data. Hence, without losing generality, and as a first step towards a more complex framework, we will consider the case where the rates are modeled using a power-law formalism. Note, however, that our approach could be easily extended in order to accommodate any other structured kinetic formalism.

### Power-law models

Using the power-law representation, the rate *v*_*r*_ is expressed as follows:

(3) $$\upsilon_{r}=\;\gamma_{r}\prod_{j/1}^{n+m}X_{j}^{f_{r,j}}\;r\;=\;1,\ldots ,\;p$$

where *γ*_*r*_ is an apparent rate constant for reaction *r*, and *f*_*r,j*_ is the kinetic order of metabolite *j* in that process. Note that this equation accounts for the effect of *n + m* metabolites (*n* dependent and *m* independent) on each reaction.

The advantage of this representation is that the same functional form represents all the rates. The reaction structure of the system will constrain the range of admissible values for some of the parameters. For example, all *γ* and *f* parameters for the substrates and catalysts of the reactions are by definition larger than zero. In addition, the values of the *f* parameters for all metabolites that are not directly involved in a given process are zero in the rate that describes the process.

By adopting such a kinetic representation, we can pose the problem of identifying the regulatory signals in a very compact mathematical form. If *X*_*j*_ is a modifier of *v*_*r*_, then the corresponding kinetic order *f*_*r,j*_ will be different from zero (positive if it is an activator, and negative if it is an inhibitor). By substituting (3) into equation (1), we get what is known as a Generalized Mass-Action (GMA) model.

(4) $${\dot{X}}_{i}=\sum_{r=1}^{p}\left(\mu_{i,r}\;\gamma_{r}\prod_{j=1}^{n+m}X_{j}^{f_{r,j}}\right)\;i=1,\ldots ,\;n$$

Note that the power-law formalism accounts for both the stoichiometry of the system (*the network structure*), and the reaction and regulatory structures (*kinetic orders*) using a single systematic nonlinear representation. This property is very important for defining a systematic way of exploring alternative regulatory signals. We will make use of this general and compact formalism in the derivation of the equations for the parameter estimation model.

### Parameter estimation in a GMA model

Given a set of experimental observations (i.e., time courses for the metabolites), our goal is to find the values of the apparent constants and kinetic orders that minimize the sum of least squared errors between the experimental data and the predicted dynamic profiles. This problem can be expressed in compact form as follows:

(5) $$\begin{array}{l} \underset{\gamma_{r},f_{r,j}}{\text{min}\;}\;\sum_{u=1}^{k}\sum_{i=1}^{n}{\left(X_{i,u}^{\text{exp}}-X_{i,u}^{\text{mod}}\right)}^{2}\\ s.t.\;{\dot{X}}_{j}=\sum_{r-1}^{p}\mu_{i,r}\upsilon_{r}\;i=1,\ldots ,n\\ \;\upsilon_{r}=\gamma_{r}\prod_{j=1}^{n+m}X_{j}^{f_{r,j}}\;r=1,\ldots ,p\;\\ \;{\dot{X}}_{i}\left(t_{0}\right)=X_{0i}\;i=1,\ldots ,n;t\;\in \left[t_{0},t_{f}\right]\\ \;X_{i,u}^{\text{mod}}=X_{i}\left(t_{u}\right)\;i=1,\ldots ,n;u\;=1,\ldots ,k \end{array}$$

where *X*_*i*_ represents the state variables (i.e., metabolite concentrations), *X*_*0i*_ their initial conditions, *X*_*i,u*_^*exp*^ denotes the experimental observations, and *X*_*i,u*_^*mod*^ are the values calculated by the dynamic model (i.e., model predictions). *i* is the index for the set of state variables whose derivatives explicitly appear in the model, *γ*_*r*_ and *f*_*r,j*_ are the parameters to be estimated, and *t*_*u*_, is the time associated with experimental point u belonging to the set *U* of observations. *k* is the total number of experimental data points and *n* is the number of time dependent variables.

Conventional parameter estimation approaches seek parameter values that minimize the approximation error assuming a given regulatory scheme (i.e., fixing some *f*_*r,j*_ to zero beforehand according to the aprioristic biochemical knowledge of the system). While this assumption simplifies the calculations, it can lead to poor approximations and hamper at the same time the discovery of new regulatory loops. In this work we introduce a rigorous and systematic parameter estimation and network identification method that makes no assumption regarding the regulatory network topology.

To model the existence of a regulatory interaction, we make use of the following disjunction:

(6) $$\begin{array}{ll} \left[\begin{array}{ll} Y_{r,j}^{-}\\ f_{r,j}\;\leq -\epsilon \; & \end{array}\right] & \underline{V}\;\left[\begin{array}{lll} Y_{r,j\;}\\ -\epsilon \leq f_{r,j}\;\leq \;\epsilon \; & & \end{array}\right]\;\underline{V}\;\left[\begin{array}{ll} Y_{r,j}^{+}\\ \epsilon \leq f_{r,j\;} & \end{array}\right]\begin{array}{l} \;j=1,\ldots ,n,\\ \;r=1,\ldots ,p \end{array}\\ & Y_{r,j}^{-},Y_{r,j},Y_{r,j\;}^{+}\in \left\{\text{True},\;\text{False}\right\} \end{array}$$

In which *Y*_*r,j*_^*-*^*,Y*_*r,j*_ and *Y*_*r,j*_^*+*^ are Boolean variables that are true if parameter *f*_*r,j*_ is negative, zero or positive, respectively, and false otherwise. ϵ is a very small parameter. Note that only one term of the disjunction can be active (i.e., exclusive disjunction), while the others must be false. Hence, if *Y*_*r,j*_ is true, metabolite *i* takes no part in velocity *r*. Conversely, if this metabolite has an influence on *r*, then *Y*_*r,j*_ is false and either *Y*_*r,j*_^*-*^ or *Y*_*r,j*_^*+*^ will be active. This disjunction can be translated into standard algebraic equations using either the big-M or convex-hull reformulations [29]. By applying the former, we get:

(7) $$\begin{array}{l} f_{r,j}\;\leq \;-\epsilon +M\left(1-y_{r,j\;}^{-}\right)\;j=1,\ldots ,\;n,r=1,\ldots ,p\\ -\epsilon -M\left(1-y_{r,j}\right)\;\leq f_{r,j}\;\leq \epsilon +M\left(1-y_{r,j\;}\right)\;j=1,\ldots ,\;n,r=1,\ldots ,p\\ f_{r,j}\;\leq \;\epsilon +M\left(1-y_{r,j\;}^{+}\right)\;j=1,\ldots ,\;n,r=1,\ldots ,p\\ y_{r,j\;}^{-}+y_{r,j}\;+y_{r,j\;}^{+}=1\;j=1,\ldots ,\;n,r=1,\ldots ,p\\ y_{r,j\;}^{-}+y_{r,j}\;+y_{r,j\;}^{+}\;\in \;\left\{0,1\right\} \end{array}$$

where Boolean variables *Y* have been replaced by auxiliary binary variables *y*. In these equations, M is a sufficiently large parameter whose value must be carefully set according to the bounds defined for the kinetic parameters.

A key issue in our approach is how to avoid overfitting. To this end, we make use of the Akaike criterion, which captures the trade-off between the number of kinetic parameters contained in the model and its ability to accurately reproduce the experimental data. If we assume that the error of the observations follows a normal distribution, the Akaike criterion takes the following mathematical form [17]:

(8) $$\begin{array}{ll} \text{AIC}= & \;k\text{log}\left(\frac{\sum_{u=1}^{k}\sum_{i=1}^{n}{\left(X_{i,u}^{\exp}-\;X_{i,u}^{\text{mod}}\right)}^{2}}{k}\right)\;\\ & +\;2\sum_{j=1}^{n}\sum_{r=1}^{p}\left(y_{r,j}^{-}+\;y_{r,j}^{+}\right)+\;C \end{array}$$

Where *AIC* denotes the value of the Akaike criterion and *C* is a constant value that does not affect the optimization. The parameter estimation problem can be finally posed in mathematical terms using the following MIDO (mixed-integer dynamic optimization) formulation:

(9) $$\begin{array}{l} \underset{\gamma_{r},f_{r,j},y_{r,j}^{-},y_{r,j},y_{r,j}^{+}}{\left(M\right)\;\text{min}\;k\text{log}}\left(\frac{\sum_{u=1}^{k}\sum_{i=1}^{n}{\left({\hat{X}}_{i,u}-\;{\bar{X}}_{i,u}\right)}^{2}}{k}\right)+2\sum_{j=1}^{n}\sum_{r=1}^{p}\left(y_{{}_{r,j}}^{-}+y_{{}_{r,j}}^{+}\right)\\ \;s.t.\;{\dot{X}}_{j}=\sum_{r-1}^{p}\mu_{i,r}\upsilon_{r}\;i=1,\ldots ,n\\ \;\upsilon_{r}=\gamma_{r}\prod_{j=1}^{n+m}X_{j}^{f_{r,j}}\;r=1,\ldots ,p\;\\ \;{\dot{X}}_{i}\left(t_{0}\right)=X_{0i}\;i=1,\ldots ,n;t\;\in \left[t_{0},t_{f}\right]\\ \;{\bar{X}}_{i,u}=X_{i}\left(t_{u}\right)\;i=1,\ldots ,n;u\;=1,\ldots ,k\\ \;f_{r,j}\;\leq \;-\epsilon +M\left(1-y_{r,j\;}^{-}\right)\;j=1,\ldots ,\;n,r=1,\ldots ,p\\ \;-\epsilon -M\left(1-y_{r,y}\right)\leq f_{r,j}\;\leq \epsilon +M\left(1-y_{r}{{}_{,}}_{j}\right)\;j=1,\ldots ,\;n,r=1,\ldots ,p\\ \;f_{r,j}\;\leq \;\epsilon +M\left(1-y_{r,j\;}^{+}\right)\;j=1,\ldots ,\;n,r=1,\ldots ,p\\ \;y_{r,j\;}^{-}+y_{r,y}+y_{r,j\;}^{+}=1\;j=1,\ldots ,\;n,r=1,\ldots ,p\\ \;y_{r,j\;}^{-}+y_{r,j}\;+y_{r,j\;}^{+}\;\in \;\left\{0,1\right\} \end{array}$$

There are different solution methods to solve this MIDO (see [25]). Without loss of generality, we propose here to reformulate this problem into an equivalent algebraic MINLP (mixed-integer nonlinear program) using orthogonal collocation on finite elements. This allows exploiting the rich optimization theory and software applications available for MINLP in the solution of the MIDO. Note that the reformulated MINLP might be nonconvex. This will give rise to multimodality (i.e., existence of multiple local optima), preventing standard gradient-based solvers from identifying the global optimum. Deterministic global optimization methods could be applied to solve the MINLP, but they might lead to large CPU times given the size and complexity of a standard dynamic problem of this type. Details on the application of deterministic global optimization methods to parameter estimation problems of small/medium size can be found elsewhere [30,31]. For the reasons given above, in this work we will solve the reformulated MINLP using local optimizers.

One important feature of our approach is that rather than calculating a single optimal solution, it identifies a set of plausible regulatory topologies by solving the model iteratively. That is, the model is first solved to identify a potential regulatory configuration represented by a binary solution (i.e., set of values of the binary variables). The model is then calculated again but this time adding the following integer cut, which excludes solutions identified so far in previous iterations from the search space:

(10) $$\begin{array}{l} \sum_{\left(r,j\right)\in \mathrm{ON}E_{\mathrm{it}}^{-}}y_{r,j}^{-\;\mathrm{it}}+\sum_{\left(r,j\right)\in \mathrm{ON}E_{\mathrm{it}}}y_{r,j}^{\;\mathrm{it}}+\sum_{\left(r,j\right)\in \mathrm{ON}E_{\mathrm{it}}^{+}}y_{r,j}^{+\;\mathrm{it}}\;\\ \;-\sum_{\left(r,j\right)\in \mathrm{ON}E_{\mathrm{it}}^{-}}y_{r,j}^{-\;\mathrm{it}}-\sum_{\left(r,j\right)\in \mathrm{ON}E_{\mathrm{it}}}y_{r,j}^{\;\mathrm{it}}-\sum_{\left(r,j\right)\in \mathrm{ON}E_{\mathrm{it}}^{+}}y_{r,t}^{+\;\mathrm{it}}\\ \;\leq \;|\;\mathrm{ON}E_{\mathrm{it}}^{-}+\mathrm{ON}E_{\mathrm{it}}+\mathrm{ON}E_{\mathrm{it}\;}^{+}|-\;1\\ \mathrm{ON}E_{\mathrm{it}\;}^{-}=\left\{\left(r,j\right)|y_{r,t}^{-\;\mathrm{it}}=1\;\mathrm{in}\;\text{the}\;\text{solution}\;\text{obtained}\;\mathrm{in}\;\text{the}\;\text{iteration}\;\mathrm{it}\;\right\}\\ \mathrm{ON}E_{\mathrm{it}}\;=\left\{\left(r,j\right)|y_{r,j}^{\;\mathrm{it}}=1\;\mathrm{in}\;\text{the}\;\text{solution}\;\text{obtained}\;\mathrm{in}\;\text{the}\;\text{iteration}\;\mathrm{it}\;\right\}\\ \mathrm{ON}E_{\mathrm{it}\;}^{+}=\left\{\left(r,j\right)|y_{r,j}^{+\;\mathrm{it}}=1\;\mathrm{in}\;\text{the}\;\text{solution}\;\text{obtained}\;\mathrm{in}\;\text{the}\;\text{iteration}\;\mathrm{it}\;\right\}\\ \text{ZER}O_{\mathrm{it}\;}^{-}=\left\{\left(r,j\right)|y_{r,j}^{-\;\mathrm{it}}=0\;\mathrm{in}\;\text{the}\;\text{solution}\;\text{obtained}\;\mathrm{in}\;\text{the}\;\text{iteration}\;\mathrm{it}\;\right\}\\ \text{ZER}O_{\mathrm{it}}\;=\left\{\left(r,j\right)|y_{r,j}^{\;\mathrm{it}}=0\;\mathrm{in}\;\text{the}\;\text{solution}\;\text{obtained}\;\mathrm{in}\;\text{the}\;\text{iteration}\;\mathrm{it}\;\right\}\\ \text{ZER}O_{\mathrm{it}\;}^{+}=\left\{\left(r,j\right)|y_{r,j}^{+\;\mathrm{it}}=0\;\mathrm{in}\;\text{the}\;\text{solution}\;\text{obtained}\;\mathrm{in}\;\text{the}\;\text{iteration}\;\mathrm{it}\;\right\} \end{array}$$

Where *ONE*_*it*_ and *ZERO*_*it*_ represent the sets of binary variables that take a value of one and zero, respectively, in iteration it of the algorithm. After adding the integer cut, the model is solved again to produce a new regulatory topology, and this procedure is repeated iteratively until a desired number of configurations is generated. Hence, the algorithm produces as output a set of potential network configurations (encoded in the values of the binary solutions) rather than a single topology. Note that these regulatory topologies show a descendant value of the Akaike performance criterion.

## Competing interests

The authors declare that they have no competing interests.

## Authors’ contributions

GGG suggested the need for the approach and provided the biological problems. AM, GGG, RA, AS and LJ developed the optimization algorithms and performed the numerical analysis. All authors evaluated the results, wrote the paper and contributed to its final form. All authors read and approved the final manuscript.

## Supplementary Material

Additional file 1: Table S1 Parameters values obtained from simulated experiments with noisy data and known regulatory structure. We generate 100 different datasets by adding random noise using a normal distribution with a standard deviation of 10%. **Table S2.** Parameter values for three experiments with noisy data and known regulatory structure (we considered three experiments and solved a total of 100 problems, replications, generated randomly with a normal distribution with a standard deviation of 10%. **Table S3.** Kinetic parameters, Akaike values and residuals corresponding to the regulatory topologies obtained by fitting an *'in silico’* experiment generated from the reference model with added noise (normal distribution with a standard deviation of 0.5% of the actual concentration value). We show the ten best cases sorted by residual value. In yellow we indicate kinetic orders that must be greater than zero as they represent effects of the substrate of the considered reaction. In green, we indicate the regulatory effects that were included in the reference model. In light red, we indicate regulatory effects that are not present in the reference model. **Table S4.** Kinetic parameters, Akaike values and residuals corresponding to the regulatory topologies obtained by fitting three *'in silico’* experiment generated from the reference model with added noise (normal distribution with a standard deviation of 0.5% of the actual concentration value). The experiments are generated from the base case by applying different perturbations in the initial concentration of *X*_*3*_. We show the ten best cases sorted by residual value. See color meaning in **Table S3**. Click here for file
