# Supplementary material for: Identification of regulatory structure and kinetic parameters of biochemical networks via mixed-integer dynamic optimization
Source: BMC Syst Biol. 2013 Oct 31;7:113. doi: 10.1186/1752-0509-7-113 (PMC3832746; doi:10.1186/1752-0509-7-113)
Supplement: Additional file 1: Table S1 — Parameters values obtained from simulated experiments with noisy data and known regulatory structure. We generate 100 different datasets by adding random noise using a normal distribution with a standard deviation of 10%. Table S2. Parameter values for three experiments with noisy data and known regulatory structure (we considered three experiments and solved a total of 100 problems, replications, generated randomly with a normal distribution with a standard deviation of 10%. Table S3. Kinetic parameters, Akaike values and residuals corresponding to the regulatory topologies obtained by fitting an 'in silico’ experiment generated from the reference model with added noise (normal distribution with a standard deviation of 0.5% of the actual concentration value). We show the ten best cases sorted by residual value. In yellow we indicate kinetic orders that must be greater than zero as they represent effects of the substrate of the considered reaction. In green, we indicate the regulatory effects that were included in the reference model. In light red, we indicate regulatory effects that are not present in the reference model. Table S4. Kinetic parameters, Akaike values and residuals corresponding to the regulatory topologies obtained by fitting three 'in silico’ experiment generated from the reference model with added noise (normal distribution with a standard deviation of 0.5% of the actual concentration value). The experiments are generated from the base case by applying different perturbations in the initial concentration of X 3 . We show the ten best cases sorted by residual value. See color meaning in Table S3. [file 1752-0509-7-113-S1.doc]

*Supplementary Material for*

**Identification of regulatory structure and kinetic parameters of biochemical networks via mixed-integer dynamic optimization**

Gonzalo Guillén-Gosálbez^1^,*, Antoni Miró^1^, Rui Alves^2^, Albert Sorribas^2^ and Laureano Jiménez^2^

Corresponding author*

Contact: gonzalo.guillen@urv.cat

**Supplementary table 1.** Parameters values obtained from simulated experiments with noisy data and known regulatory structure. We generate 100 different datasets by adding random noise using a normal distribution with a standard deviation of 10%. In the estimation task, the non-zero parameters are indicated and all the other parameters are set to zero.

| Profile | f_13_ | f_21_ | f_32_ | f_41_ | f_53_ | f_54_ | f_64_ | Residual |
| --- | --- | --- | --- | --- | --- | --- | --- | --- |
| 1 | -0.14 | 0.26 | 0.44 | 0.04 | 0 | -0.06 | 0.13 | 1.88 |
| 2 | -0.27 | 0.47 | 1 | 0 | 0.26 | 0.04 | 0.07 | 1.67 |
| 3 | -0.84 | 0.4 | 0.64 | 0.9 | 0.42 | 0.1 | 1 | 1.68 |
| 4 | -0.79 | 0.29 | 0.41 | 1 | 0.12 | -0.12 | 1 | 2.29 |
| 5 | -0.77 | 0.58 | 1 | 0.88 | 0 | 0.22 | 1 | 1.81 |
| 6 | 0.62 | 0.12 | 0.2 | 0 | 0.41 | -1 | 0.93 | 2.14 |
| 7 | -0.65 | 0.33 | 0.5 | 0.27 | 0.97 | 0.21 | 0.5 | 1.42 |
| 8 | -0.45 | 0.4 | 0.62 | 0 | 0.12 | 0.27 | 0.28 | 1.78 |
| 9 | -0.42 | 0.66 | 1 | 0.19 | 0 | -0.63 | 0.35 | 1.93 |
| 10 | -1 | 0.79 | 0.79 | 0 | 0.73 | 0.58 | 1 | 1.64 |
| 11 | -0.64 | 0.86 | 1 | 0 | 0.49 | 0.61 | 1 | 1.89 |
| 12 | -0.36 | 0.33 | 0.41 | 0 | 0.3 | 0.23 | 0.38 | 1.19 |
| 13 | -0.39 | 0.26 | 0.47 | 0 | 0 | 0.07 | 1 | 2.01 |
| 14 | -0.42 | 0.12 | 0.18 | 0.58 | 0 | -0.15 | 0.58 | 2.06 |
| 15 | 0.25 | 0.02 | 0.53 | 0 | 1 | -0.16 | 0.02 | 5.62 |
| 16 | -0.54 | 0.26 | 0.39 | 0.41 | 0.31 | 0.06 | 0.54 | 1.11 |
| 17 | -0.27 | 0.11 | 0.38 | 0.45 | 0 | -0.2 | 0.72 | 1.6 |
| 18 | 0.37 | 0.07 | 0.11 | 0 | 1 | 0.12 | 1 | 1.53 |
| 19 | -1 | 0.71 | 1 | 0.13 | 0.95 | 0.7 | 0.31 | 1.17 |
| 20 | -0.49 | 0.26 | 0.41 | 0.7 | 0.22 | 0.08 | 1 | 2.03 |
| 21 | -0.43 | 0.84 | 1 | 0 | 0.14 | 0.11 | 0.06 | 2.25 |
| 22 | 0.56 | 0.14 | 0.06 | 0 | 1 | -1 | 0.87 | 2.52 |
| 23 | -0.31 | 0.57 | 1 | 0 | 0 | -0.07 | 1 | 1.93 |
| 24 | -1 | 0.5 | 0.89 | 0.88 | 0.23 | 0.13 | 1 | 1.27 |
| 25 | -0.22 | 0.22 | 0.28 | 0.31 | 0.13 | 0.04 | 1 | 1.28 |
| 26 | -0.3 | 0.61 | 1 | 0 | 0.42 | 0.03 | 0 | 2.85 |
| 27 | -0.05 | 1 | 0.67 | 1 | 0 | -0.12 | 0.16 | 37.74 |
| 28 | -0.76 | 0.72 | 1 | 0 | 0.36 | 0.58 | 1 | 1.89 |
| 29 | -0.03 | 0.91 | 1 | 0 | 0.88 | -0.51 | 0.05 | 2.33 |
| 30 | -0.75 | 0.22 | 0.53 | 0.98 | 0 | -0.1 | 1 | 2.24 |
| 31 | -0.11 | 0.19 | 0.3 | 0 | 0.11 | -0.06 | 0.31 | 1.71 |
| 32 | -0.53 | 0.23 | 0.37 | 0.67 | 0.12 | -0.08 | 1 | 2.12 |
| 33 | -0.84 | 0.72 | 1 | 0.1 | 1 | 0.77 | 0.44 | 1.3 |
| 34 | -0.77 | 0.24 | 0.46 | 0.95 | 0.18 | -0.05 | 1 | 2.12 |
| 35 | -0.63 | 0.34 | 0.55 | 0.39 | 0.46 | 0.26 | 0.73 | 2.66 |
| 36 | -0.55 | 0.77 | 1 | 0 | 0.63 | 0.39 | 0.07 | 1.38 |
| 37 | -0.33 | 0.21 | 0.41 | 0 | 0.12 | 0.07 | 1 | 1.94 |
| 38 | -0.17 | 0.27 | 0.4 | 0.18 | 0.02 | -0.01 | 0.26 | 1.61 |
| 39 | -0.6 | 0.21 | 0.53 | 1 | 0 | -0.04 | 1 | 1.75 |
| 40 | -0.66 | 0.32 | 0.54 | 0.89 | 0.16 | 0.07 | 1 | 1.75 |
| 41 | -0.34 | 0.18 | 0.44 | 0.66 | 0 | -0.03 | 1 | 2.07 |
| 42 | -0.39 | 0.31 | 0.31 | 0.46 | 0.16 | -0.04 | 1 | 1.34 |
| 43 | -0.36 | 0.23 | 0.27 | 0 | 0.18 | -1 | 1 | 2.81 |
| 44 | -1 | 0.48 | 0.65 | 0.87 | 0.4 | 0.21 | 1 | 1.1 |
| 45 | -0.9 | 0.03 | 0.02 | 1 | 1 | 0.06 | 1 | 18.53 |
| 46 | -0.98 | 0.95 | 1 | 0.28 | 0.12 | 0.35 | 0.38 | 1.04 |
| 47 | -0.28 | 0.49 | 1 | 0 | 0.35 | 0.16 | 0.05 | 2.06 |
| 48 | -0.38 | 0.24 | 0.45 | 0 | 1 | 0.54 | 1 | 1.98 |
| 49 | -0.51 | 0.29 | 0.41 | 0.59 | 0.29 | 0.1 | 1 | 1.48 |
| 50 | -0.17 | 0.2 | 0.33 | 0 | 0.89 | 0.83 | 0.49 | 2.47 |
| 51 | -0.94 | 0.54 | 0.82 | 0.62 | 0.58 | 0.45 | 1 | 1.29 |
| 52 | -0.84 | 0.3 | 0.43 | 1 | 0.2 | -0.24 | 1 | 2.35 |
| 53 | -0.36 | 1 | 0.94 | 0.09 | 0.38 | -0.25 | 0.03 | 2.67 |
| 54 | -0.83 | 0.61 | 1 | 0.71 | 0.12 | 0.22 | 1 | 1.64 |
| 55 | -1 | 0.57 | 1 | 0.47 | 1 | 0.75 | 0.78 | 1.16 |
| 56 | -0.3 | 0.36 | 0.61 | 0 | 0.92 | 1 | 0.3 | 1.15 |
| 57 | -0.43 | 0.78 | 1 | 0 | 0.53 | 1 | 0.94 | 1.71 |
| 58 | -0.22 | 0.43 | 0.63 | 0 | 0.89 | 1 | 0.28 | 2.04 |
| 59 | -0.37 | 0.28 | 0.53 | 0.04 | 0.44 | 0.32 | 0.35 | 1.64 |
| 60 | -0.38 | 0.52 | 1 | 0 | 0 | 0.2 | 0.1 | 1.78 |
| 61 | -0.91 | 0.6 | 1 | 0.27 | 0.02 | 0.19 | 0.23 | 1.79 |
| 62 | -0.83 | 0.55 | 0.62 | 1 | 0.13 | 0 | 1 | 1.49 |
| 63 | -0.52 | 0.26 | 0.42 | 0 | 0.26 | -1 | 1 | 2.23 |
| 64 | -1 | 0.71 | 1 | 0.26 | 0.16 | 0.19 | 0.24 | 1.85 |
| 65 | -0.42 | 0.37 | 0.48 | 0.18 | 0.03 | 0.12 | 0.56 | 1.74 |
| 66 | -0.36 | 0.25 | 0.43 | 0 | 0.13 | 0.04 | 0.28 | 1.11 |
| 67 | -0.87 | 0.74 | 1 | 0.23 | 0.54 | 0.25 | 0.18 | 1.45 |
| 68 | 0 | 0.59 | 1 | 0 | 0.09 | -0.12 | 0 | 9.04 |
| 69 | -1 | 0.57 | 0.9 | 1 | 1 | 0.03 | 0.23 | 1.93 |
| 70 | -1 | 0.64 | 1 | 0.88 | 0.54 | 0.32 | 1 | 1.62 |
| 71 | -1 | 0.73 | 1 | 0.7 | 0.16 | 0.18 | 1 | 1.73 |
| 72 | -0.38 | 0.24 | 0.39 | 0 | 0.33 | 0.16 | 0.46 | 1.42 |
| 73 | -0.24 | 0.25 | 0.39 | 0 | 0.23 | 0.12 | 0.37 | 1.93 |
| 74 | -0.36 | 0.08 | 0.17 | 1 | 0 | -0.2 | 1 | 2.39 |
| 75 | -0.35 | 0.23 | 0.38 | 0 | 0.09 | 0.09 | 0.34 | 1.27 |
| 76 | -1 | 0.65 | 1 | 0.6 | 0.7 | 0.46 | 1 | 1.69 |
| 77 | -1 | 0.76 | 1 | 0.24 | 0.54 | 0.08 | 0.42 | 1.41 |
| 78 | -0.65 | 0.51 | 1 | 0.39 | 0.45 | 0.19 | 0.44 | 2.02 |
| 79 | 0.32 | 0 | 0.13 | 0.01 | 0.37 | -0.01 | 0.13 | 4.22 |
| 80 | -0.54 | 0.3 | 0.51 | 0.48 | 0.18 | 0.17 | 1 | 1.31 |
| 81 | -1 | 0.75 | 1 | 0.33 | 0.23 | 0.33 | 0.49 | 1.67 |
| 82 | -0.54 | 0.2 | 0.39 | 0.97 | 0 | -0.09 | 1 | 1.67 |
| 83 | -1 | 0.95 | 1 | 0.4 | 1 | 1 | 1 | 2.12 |
| 84 | -1 | 0.72 | 1 | 0.32 | 0.34 | 0.21 | 0.3 | 1.71 |
| 85 | -0.63 | 0.32 | 0.54 | 0.88 | 0.12 | 0.07 | 1 | 2.77 |
| 86 | -1 | 0.88 | 1 | 0.25 | 0.47 | 0.3 | 0.19 | 1.82 |
| 87 | -1 | 0.64 | 0.87 | 0 | 0.17 | 0.2 | 1 | 1.75 |
| 88 | 0 | 0 | 0.17 | 0.01 | 0.4 | -0.12 | 0 | 12.4 |
| 89 | -0.25 | 0.2 | 0.3 | 0 | 0.16 | -0.01 | 0.86 | 0.94 |
| 90 | -1 | 0.58 | 0.81 | 0.01 | 1 | 0.37 | 0.21 | 2.13 |
| 91 | -0.67 | 0.7 | 1 | 0.01 | 1 | 1 | 0.44 | 1.44 |
| 92 | -0.56 | 0.6 | 1 | 0.17 | 0.09 | 0.14 | 0.17 | 2.12 |
| 93 | -0.15 | 0.22 | 0.32 | 0 | 0.11 | -0.01 | 0.92 | 1.94 |
| 94 | -0.27 | 0.19 | 0.28 | 0.89 | 0 | -0.09 | 1 | 2.09 |
| 95 | -0.65 | 0.26 | 0.65 | 1 | 0.19 | 0.12 | 1 | 1.58 |
| 96 | 0.4 | 0.21 | 0.14 | 0.23 | 1 | 0.02 | 0 | 5.62 |
| 97 | -0.42 | 0.47 | 0.5 | 0 | 0.64 | 0.82 | 0.46 | 1.07 |
| 98 | -0.75 | 0.29 | 0.43 | 1 | 0 | -0.12 | 1 | 1.56 |
| 99 | -0.33 | 0.24 | 0.28 | 0 | 0.26 | -1 | 0 | 3.19 |
| 100 | -0.15 | 0.16 | 0.39 | 0 | 0.19 | 0.12 | 0.69 | 1.57 |

**Supplementary table 2.** Parameter values for three experiments with noisy data and known regulatory structure (we considered three experiments and solved a total of 100 problems, replications, generated randomly with a normal distribution with a standard deviation of 10%.

| Profile | f_12_ | f_14_ | f_23_ | f_31_ | f_35_ | f_45_ | f_46_ | Residual |
| --- | --- | --- | --- | --- | --- | --- | --- | --- |
| 1 | 0.33 | 0.64 | 0.42 | -0.67 | 0.49 | 0.05 | 1 | 6.96 |
| 2 | 0.9 | 0 | 1 | -0.64 | 0.66 | -0.95 | 1 | 7.1 |
| 3 | 0.49 | 0.38 | 0.73 | -0.62 | 0.3 | 0.22 | 0.53 | 5.39 |
| 4 | 0.69 | 0.26 | 1 | -0.92 | 0.4 | 0.34 | 0.58 | 4.89 |
| 5 | 0.31 | 0.71 | 0.54 | -0.49 | 0.24 | 0.13 | 1 | 5.58 |
| 6 | 0 | 0.01 | 0.2 | 0.18 | 0.35 | -0.03 | 0.06 | 22.51 |
| 7 | 0.58 | 0.27 | 1 | -0.64 | 0.69 | 0.26 | 0.44 | 6.14 |
| 8 | 0.51 | 0.12 | 0.83 | -0.72 | 0.46 | 0.17 | 0.17 | 5.02 |
| 9 | 0.6 | 0.59 | 0.8 | -0.79 | 0.64 | 0.08 | 0.86 | 5.96 |
| 10 | 0.56 | 0 | 1 | -0.57 | 0.26 | -0.46 | 1 | 8.3 |
| 11 | 0.31 | 0.59 | 0.54 | -0.52 | 0.16 | 0.12 | 0.74 | 7.04 |
| 12 | 0.55 | 0.5 | 1 | -1 | 0.69 | 0.63 | 1 | 6.19 |
| 13 | 0.49 | 0.23 | 0.6 | -0.66 | 0.6 | 0.18 | 0.29 | 6.58 |
| 14 | 0.61 | 0.34 | 1 | -0.61 | 0.42 | -0.03 | 0.48 | 6.82 |
| 15 | 0.52 | 0 | 0.81 | -0.52 | 0.19 | 0.26 | 0.52 | 7.37 |
| 16 | 0.68 | 0.16 | 0.98 | -0.72 | 0.87 | 0.52 | 0.29 | 5.65 |
| 17 | 0.56 | 0.7 | 1 | -0.87 | 0.41 | 0.25 | 1 | 4.26 |
| 18 | 0.4 | 0.74 | 0.69 | -0.78 | 0.3 | 0.11 | 1 | 5.76 |
| 19 | 0.26 | 0.52 | 0.48 | -0.62 | 0.33 | 0 | 0.85 | 7.54 |
| 20 | 0.46 | 0.61 | 0.77 | -0.78 | 0.38 | 0.23 | 1 | 5.54 |
| 21 | 0.39 | 0.72 | 0.62 | -0.72 | 0.22 | 0.05 | 1 | 5.59 |
| 22 | 1 | 0 | 1 | 0.14 | 1 | -1 | 0 | 36.61 |
| 23 | 0.17 | 0.05 | 0.3 | 1 | 0 | -1 | 1 | 18.13 |
| 24 | 0.6 | 0.08 | 0.94 | -0.91 | 0.52 | -0.06 | 0.66 | 8.37 |
| 25 | 0.55 | 0 | 0.84 | -0.9 | 0.57 | -0.12 | 0.65 | 7.66 |
| 26 | 0.46 | 0.68 | 0.67 | -0.78 | 0.48 | 0.12 | 1 | 5.07 |
| 27 | 0.2 | 0 | 0.04 | 1 | 0.99 | -0.05 | 0.02 | 43.47 |
| 28 | 0.46 | 0 | 0.75 | 1 | 0 | -1 | 1 | 18 |
| 29 | 0.32 | 0.13 | 0.43 | -0.5 | 0.29 | 0.13 | 0.3 | 5.73 |
| 30 | 0.5 | 0.39 | 0.72 | -0.76 | 0.45 | 0.33 | 0.53 | 6.61 |
| 31 | 0.31 | 1 | 0.36 | -1 | 0 | -1 | 0.44 | 74.29 |
| 32 | 0.31 | 0.33 | 0.49 | -0.45 | 0.14 | 0.1 | 1 | 6.32 |
| 33 | 0.64 | 0.22 | 1 | -0.9 | 0.7 | 0.47 | 0.46 | 5.23 |
| 34 | 0.72 | 0.39 | 1 | -0.89 | 0.82 | 0.3 | 0.47 | 5.6 |
| 35 | 0.72 | 0.09 | 1 | -0.74 | 0.64 | 0.47 | 0.19 | 5.71 |
| 36 | 0.44 | 0.65 | 0.62 | -0.77 | 0.45 | 0.19 | 1 | 6.93 |
| 37 | 0.75 | 0.01 | 0.99 | -0.59 | 0.61 | 0.2 | 0.03 | 7.02 |
| 38 | 0.6 | 0.12 | 1 | -0.57 | 0.49 | 0.25 | 0.18 | 5.35 |
| 39 | 0.32 | 0.63 | 0.53 | -0.61 | 0.15 | -0.02 | 1 | 4.77 |
| 40 | 0.6 | 0.04 | 0.86 | -0.73 | 0.39 | 0.36 | 0.61 | 8.59 |
| 41 | 0.54 | 0.67 | 0.92 | -0.95 | 0.54 | 0.18 | 1 | 6.05 |
| 42 | 0.51 | 0.6 | 0.73 | -0.87 | 0.55 | 0.04 | 1 | 4.48 |
| 43 | 0.61 | 0.1 | 1 | -0.58 | 0.74 | 0.35 | 0.15 | 4.71 |
| 44 | 0.39 | 0.63 | 0.64 | -0.78 | 0.27 | -0.02 | 1 | 5.15 |
| 45 | 0.7 | 0 | 1 | -0.59 | 0.6 | 0.89 | 0.55 | 5.71 |
| 46 | 0.64 | 0.35 | 1 | -0.96 | 0.48 | 0.21 | 1 | 7.81 |
| 47 | 0.35 | 0.33 | 0.52 | -0.59 | 0.29 | 0.02 | 0.71 | 5.85 |
| 48 | 0.46 | 0.18 | 0.69 | -0.65 | 0.36 | 0.16 | 0.37 | 4.94 |
| 49 | 0.66 | 0.08 | 1 | -0.59 | 0.72 | 0.2 | 0.15 | 5.39 |
| 50 | 0.62 | 0 | 1 | -0.96 | 0.67 | 0.33 | 0.45 | 5.71 |
| 51 | 0 | 1 | 0.42 | 0.38 | 1 | -0.13 | 1 | 19.11 |
| 52 | 0.62 | 0.2 | 1 | -0.83 | 0.66 | 0.29 | 0.32 | 6.27 |
| 53 | 0 | 0 | 1 | 0.22 | 1 | -1 | 0 | 27.25 |
| 54 | 1 | 0 | 1 | 1 | 1 | -1 | 0.02 | 21.33 |
| 55 | 0.52 | 0.09 | 0.77 | -0.64 | 0.46 | 0.29 | 0.39 | 4.66 |
| 56 | 0.46 | 0.17 | 0.67 | -0.67 | 0.4 | 0.32 | 0.42 | 5.94 |
| 57 | 0.3 | 0.11 | 0.49 | -0.39 | 0.19 | 0.13 | 0.38 | 5.61 |
| 58 | 0.44 | 0 | 0.53 | -0.44 | 0.22 | 0.23 | 0.83 | 5.24 |
| 59 | 0.41 | 0.63 | 0.58 | -0.77 | 0.43 | 0.26 | 1 | 7.23 |
| 60 | 0.07 | 0 | 0 | 0.4 | 1 | 0.01 | 0.01 | 42.55 |
| 61 | 0.17 | 0.08 | 0.04 | 1 | 0 | -0.37 | 1 | 33.1 |
| 62 | 0.61 | 0.59 | 1 | -0.92 | 0.37 | 0.17 | 1 | 4.71 |
| 63 | 0.63 | 0 | 0.97 | -0.79 | 0.58 | 0 | 0.02 | 7 |
| 64 | 0.55 | 0.3 | 0.9 | -0.68 | 0.68 | 0.57 | 0.53 | 7.72 |
| 65 | 0.37 | 0.67 | 0.52 | -0.68 | 0.38 | 0.16 | 1 | 6.53 |
| 66 | 0.44 | 0.7 | 0.71 | -0.76 | 0.38 | 0.16 | 1 | 6.25 |
| 67 | 0.49 | 0.37 | 1 | -0.7 | 0.33 | 0.17 | 0.59 | 7.31 |
| 68 | 0.27 | 0.69 | 0.4 | -0.45 | 0.14 | 0.07 | 1 | 6.1 |
| 69 | 0.56 | 0.23 | 0.82 | -0.69 | 0.34 | 0.08 | 0.39 | 5.05 |
| 70 | 0.63 | 0 | 0.94 | -0.38 | 0.49 | 0.2 | 0.06 | 4.82 |
| 71 | 0.57 | 0.06 | 0.96 | -0.63 | 0.52 | 0.19 | 0.2 | 5.71 |
| 72 | 0.59 | 0.2 | 0.92 | -0.71 | 0.56 | 0.61 | 0.48 | 6.15 |
| 73 | 0.53 | 0.3 | 0.95 | -0.8 | 0.49 | 0.38 | 0.51 | 5.75 |
| 74 | 0.63 | 0.09 | 0.96 | -0.5 | 0.48 | 0.04 | 0.16 | 5.93 |
| 75 | 0.63 | 0.49 | 1 | -1 | 0.7 | 0.26 | 0.81 | 4.41 |
| 76 | 0.42 | 0.24 | 0.59 | -0.58 | 0.34 | 0.19 | 0.37 | 5.86 |
| 77 | 0.64 | 0.32 | 1 | -0.88 | 0.62 | 0.23 | 0.39 | 7.05 |
| 78 | 0.21 | 0.02 | 0.37 | -0.01 | 0.01 | -0.01 | 0 | 30.56 |
| 79 | 0.56 | 0.32 | 0.92 | -0.86 | 0.39 | 0.37 | 0.44 | 5.66 |
| 80 | 0.06 | 0 | 0.09 | 1 | 0.3 | -1 | 0.87 | 15.24 |
| 81 | 1 | 0.01 | 0.3 | 0.53 | 1 | 0.29 | 0.01 | 22.46 |
| 82 | 0.59 | 0.25 | 1 | -0.69 | 0.31 | 0.23 | 0.53 | 6.88 |
| 83 | 0.32 | 0.24 | 0.59 | -0.44 | 0.28 | 0.31 | 0.56 | 5.19 |
| 84 | 0.48 | 0 | 0.82 | -0.59 | 0.29 | 0.19 | 0.23 | 5.8 |
| 85 | 0.47 | 0.7 | 0.77 | -0.92 | 0.34 | 0.03 | 1 | 6.19 |
| 86 | 0.68 | 0 | 1 | -1 | 0.54 | 0.28 | 0.8 | 5.08 |
| 87 | 0.31 | 0.41 | 0.44 | -0.51 | 0.26 | 0.03 | 0.71 | 6.21 |
| 88 | 0 | 1 | 0.9 | -1 | 0 | -1 | 1 | 34.87 |
| 89 | 0.36 | 0.35 | 0.68 | -0.64 | 0.33 | 0.2 | 0.54 | 5.18 |
| 90 | 0.56 | 0.09 | 1 | -0.69 | 0.46 | 0.29 | 0.3 | 5.72 |
| 91 | 0.14 | 0.04 | 0.04 | 1 | 0 | -0.41 | 1 | 35.86 |
| 92 | 0.4 | 0.68 | 0.71 | -0.62 | 0.35 | 0.2 | 1 | 5.77 |
| 93 | 0.65 | 0.08 | 0.98 | -0.86 | 0.29 | 0.27 | 0.54 | 6.52 |
| 94 | 0.73 | 0.25 | 1 | -1 | 0.52 | 0.12 | 0.39 | 6.48 |
| 95 | 0.01 | 0.51 | 0.48 | 1 | 0.42 | -0.15 | 1 | 26.25 |
| 96 | 0.46 | 0.18 | 0.63 | -0.51 | 0.54 | 0.16 | 0.32 | 6.32 |
| 97 | 0.42 | 0.31 | 0.5 | -0.54 | 0.32 | 0.01 | 0.5 | 5.55 |
| 98 | 0 | 0.02 | 0.32 | -1 | 0.38 | -1 | 0.32 | 39.4 |
| 99 | 0.63 | 0.11 | 0.98 | -0.66 | 0.85 | 0.48 | 0.35 | 6.14 |
| 100 | 0.55 | 0 | 0.71 | -0.87 | 0.5 | 0.02 | 0.34 | 6.31 |

**Supplementary table 3.** Kinetic parameters, Akaike values and residuals corresponding to the regulatory topologies obtained by fitting an *‘in silico’* experiment generated from the reference model with added noise (normal distribution with a standard deviation of 0.5% of the actual concentration value). We show the ten best cases sorted by residual value. In yellow we indicate kinetic orders that must be greater than zero as they represent effects of the substrate of the considered reaction. In green, we indicate the regulatory effects that were included in the reference model. In light red, we indicate regulatory effects that are not present in the reference model.

| Topology |  | *v*_1_ | *v*_2_ | *v*_3_ | *v*_4_ | *v*_5_ | *v*_6_ | Akaike | Residual |
| --- | --- | --- | --- | --- | --- | --- | --- | --- | --- |
| 1 | X_1_ | - | 0.59 | - | 0.4 | 0.27 | - | -176.18 | 0.00223 |
|  | X_2_ | - | - | 1 | - | - | - |  |  |
|  | X_3_ | -0.82 | - | - | - | 0.88 | - |  |  |
|  | X_4_ | - | - | -0.08 | - | - | 0.62 |  |  |
| 2 | X_1_ | 0.08 | 0.55 | - | 0.31 | - | - | -171.12 | 0.00283 |
|  | X_2_ | - | - | 0.83 | - | - | - |  |  |
|  | X_3_ | -0.63 | - | - | - | 0.62 | - |  |  |
|  | X_4_ | - | - | - | - | 0.3 | 0.46 |  |  |
| 3 | X_1_ | -0.01 | 0.52 | - | 0.35 | - | - | -168.86 | 0.00316 |
|  | X_2_ | - | - | 0.77 | - | - | - |  |  |
|  | X_3_ | -0.76 | - | - | - | 0.49 | - |  |  |
|  | X_4_ | - | - | - | - | 0.21 | 0.6 |  |  |
| 4 | X_1_ | -0.06 | 0.38 | - | 0.22 | - | - | -168.42 | 0.00322 |
|  | X_2_ | - | - | 0.55 | - | 0.07 | - |  |  |
|  | X_3_ | -0.58 | - | - | - | 0.28 | - |  |  |
|  | X_4_ | - | - | - | - | - | 0.41 |  |  |
| 5 | X_1_ | - | 0.48 | - | 0.36 | 0.31 | - | -167.15 | 0.00342 |
|  | X_2_ | - | - | 0.72 | - | - | - |  |  |
|  | X_3_ | -0.72 | - | - | - | 0.76 | - |  |  |
|  | X_4_ | - | - | 0.02 | - | - | 0.62 |  |  |
| 6 | X_1_ | - | 0.33 | - | 0.27 | - | - | -168.45 | 0.00354 |
|  | X_2_ | - | - | 0.48 | - | - | - |  |  |
|  | X_3_ | -0.5 | - | - | - | 0.28 | -0.07 |  |  |
|  | X_4_ | - | - | - | - | - | 0.4 |  |  |
| 7 | X_1_ | - | 0.46 | - | 0.47 | - | - | -166.42 | 0.00355 |
|  | X_2_ | - | - | 0.59 | - | - | - |  |  |
|  | X_3_ | -0.74 | - | -0.12 | - | 0.27 | - |  |  |
|  | X_4_ | - | - | - | - | 0.18 | 0.8 |  |  |
| 8 | X_1_ | - | 0.41 | - | 0.28 | 0.2 | - | -166.06 | 0.0036 |
|  | X_2_ | - | - | 0.59 | - | - | - |  |  |
|  | X_3_ | -0.6 | - | -0.06 | - | 0.54 | - |  |  |
|  | X_4_ | - | - | - | - | - | 0.52 |  |  |
| 9 | X_1_ | - | 0.47 | - | 0.14 | - | - | -165.58 | 0.0037 |
|  | X_2_ | - | - | 0.6 | - | - | - |  |  |
|  | X_3_ | -0.56 | - | - | - | 0.38 | - |  |  |
|  | X_4_ | - | - | 0.08 | - | 0.27 | 0.27 |  |  |
| 10 | X_1_ | - | 0.7 | - | 0.41 | 0.26 | - | -165.57 | 0.00369 |
|  | X_2_ | - | - | 0.89 | - | - | - |  |  |
|  | X_3_ | -0.91 | - | - | - | 0.87 | - |  |  |
|  | X_4_ | - | -0.15 | - | - | - | 0.64 |  |  |

**Supplementary table 4.** Kinetic parameters, Akaike values and residuals corresponding to the regulatory topologies obtained by fitting three *‘in silico’* experiment generated from the reference model with added noise (normal distribution with a standard deviation of 0.5% of the actual concentration value). The experiments are generated from the base case by applying different perturbations in the initial concentration of *X_3_*. We show the ten best cases sorted by residual value. In yellow we indicate kinetic orders that must be greater than zero as they represent effects of the substrate of the considered reaction. In green, we indicate the regulatory effects that were included in the reference model. In light red, we indicate regulatory effects that are not present in the reference model.

| Topology |  | *v*_1_ | *v*_2_ | *v*_3_ | *v*_4_ | *v*_5_ | *v*_6_ | Akaike | Residual |
| --- | --- | --- | --- | --- | --- | --- | --- | --- | --- |
| 1 | X_1_ | - | 0.52 | - | 0.3 | - | - | -483.49 | 0.0137 |
|  | X_2_ | - | - | 0.77 | -0.01 | - | - |  |  |
|  | X_3_ | -0.74 | - | - | - | 0.54 | - |  |  |
|  | X_4_ | - | - | - | - | 0.2 | 0.5 |  |  |
| 2 | X_1_ | - | 0.48 | - | 0.38 | - | - | -480.62 | 0.0143 |
|  | X_2_ | -0.01 | - | 0.73 | - | - | - |  |  |
|  | X_3_ | -0.75 | - | - | - | 0.49 | - |  |  |
|  | X_4_ | - | - | - | - | 0.2 | 0.65 |  |  |
| 3 | X_1_ | - | 0.49 | - | 0.35 | - | - | -480.53 | 0.01431 |
|  | X_2_ | - | - | 0.74 | - | - | - |  |  |
|  | X_3_ | -0.75 | - | 0.02 | - | 0.5 | - |  |  |
|  | X_4_ | - | - | - | - | 0.21 | 0.61 |  |  |
| 4 | X_1_ | - | 0.52 | - | 0.31 | - | - | -479.19 | 0.01463 |
|  | X_2_ | - | - | 0.78 | - | - | 0.02 |  |  |
|  | X_3_ | -0.76 | - | - | - | 0.54 | - |  |  |
|  | X_4_ | - | - | - | - | 0.2 | 0.5 |  |  |
| 5 | X_1_ | - | 0.52 | - | 0.31 | - | - | -484.84 | 0.0147 |
|  | X_2_ | - | - | 0.8 | - | - | - |  |  |
|  | X_3_ | -0.74 | - | - | - | 0.57 | - |  |  |
|  | X_4_ | - | - | - | - | 0.21 | 0.54 |  |  |
| 6 | X_1_ | - | 0.52 | - | 0.3 | - | - | -478.44 | 0.0148 |
|  | X_2_ | 0.01 | - | 0.8 | - | - | - |  |  |
|  | X_3_ | -0.75 | - | - | - | 0.52 | - |  |  |
|  | X_4_ | - | - | - | - | 0.21 | 0.54 |  |  |
| 7 | X_1_ | - | 0.53 | -0.01 | 0.29 | - | - | -477.18 | 0.0151 |
|  | X_2_ | - | - | 0.79 | - | - | - |  |  |
|  | X_3_ | -0.72 | - | - | - | 0.57 | - |  |  |
|  | X_4_ | - | - | - | - | 0.22 | 0.45 |  |  |
| 8 | X_1_ | - | 0.5 | - | 0.32 | - | - | -476.31 | 0.0153 |
|  | X_2_ | - | - | 0.76 | - | - | - |  |  |
|  | X_3_ | -0.72 | - | 0.04 | - | 0.55 | - |  |  |
|  | X_4_ | - | - | - | - | 0.21 | 0.55 |  |  |
| 9 | X_1_ | - | 0.53 | - | 0.33 | - | 0.01 | -475.8 | 0.01544 |
|  | X_2_ | - | - | 0.77 | - | - | - |  |  |
|  | X_3_ | -0.75 | - | - | - | 0.53 | - |  |  |
|  | X_4_ | - | - | - | - | 0.19 | 0.54 |  |  |
| 10 | X_1_ | - | 0.54 | - | 0.29 | - | - | -475.54 | 0.0155 |
|  | X_2_ | - | - | 0.8 | - | - | -0.01 |  |  |
|  | X_3_ | -0.73 | - | - | - | 0.54 | - |  |  |
|  | X_4_ | - | - | - | - | 0.22 | 0.48 |  |  |
